# Supplementary material for: Content-rich biological network constructed by mining PubMed abstracts
Source: BMC Bioinformatics. 2004 Oct 8;5:147. doi: 10.1186/1471-2105-5-147 (PMC528731; doi:10.1186/1471-2105-5-147)
Supplement: Additional File 2 — The original results of the above study (non-essential files are deleted to keep the file size under the limit set by BMC bioinformatics). [file 1471-2105-5-147-S2.bz2 › chilibotAdditionalFile2/dip10/608ID1701053E10990/html/CSF3R_CSF3.html]

 


 **CSF3R** and **CSF3** 
  
Found 215 abstracts in PubMed, retrieved 10.  
 

 What does Google say? 
 PDF only 
| .edu only 

---

**Interactive relationship** (e.g. stimulation, inhibition, etc)

**Stimulatory relationship**- Granulocyte colony stimulating factor  [ **CSF3** ]  G CSF  [ **CSF3** ]  induces proliferation, maturation, and functional activities of myeloid progenitors and mature neutrophils through a specific receptor, the G CSF  [ **CSF3** ]  R  [ **CSF3R** ] .  Ref: 12586631 Blood, 2003
**Neutral relationship**- G CSF  [ **CSF3** ]  activates a receptor of the hematopoietin receptor superfamily, the G CSF  [ **CSF3** ]  receptor G CSF  [ **CSF3** ]  R  [ **CSF3R** ] , which subsequently triggers multiple signaling mechanisms.  Ref: 12687405 Rev Physiol Biochem Pharmacol< MedlineTA>Rev Physiol Biochem Pharmacol, 2003
- G CSF  [ **CSF3** ]  affects these cells after binding to its specific receptor, G CSF  [ **CSF3** ]  R  [ **CSF3R** ] , which exists in seven isoforms.  Ref: 12660437 Biol Neonate, 2003

**Non-interactive relationship** (e.g. studied together, co-existance, homology, etc.)

- Here we review how these mechanisms contribute to the specific responses of hematopoietic cells to G CSF  [ **CSF3** ]  and how perturbations in the function of the G CSF  [ **CSF3** ]  R  [ **CSF3R** ]  are implicated in various types of myeloid disease.  Ref: 12687405 Rev Physiol Biochem Pharmacol< MedlineTA>Rev Physiol Biochem Pharmacol, 2003
- Real time PCR analysis revealed that TPT augmented the expression not only of CD18 but also of components of superoxide generating NADPH oxidase, p47phox, 2.7 fold, and p67phox, 2.0 fold, and of granulocyte colony stimulating factor  [ **CSF3** ]  receptor  [ **CSF3R** ]  G CSFR, 3.0 fold.  Ref: 12788061 Biochem Biophys Res Commun, 2003
- Messenger RNA expression of granulocyte colony stimulating factor  [ **CSF3** ]  receptor  [ **CSF3R** ]  isoforms in the fetus.  Ref: 12660437 Biol Neonate, 2003
- Long term follow up of granulocyte colony stimulating factor  [ **CSF3** ]  receptor  [ **CSF3R** ]  mutations in patients with severe congenital neutropenia implications for leukaemogenesis and therapy.  Ref: 12588357 Br J Haematol, 2003
- We evaluated the involvement of Syk kinase, essential in mediating phagocytic signals by Fcgamma receptors, in G CSF  [ **CSF3** ]  induced phagocytosis, using murine myeloid 32D cells transfected with wild type WT human G CSF  [ **CSF3** ]  R  [ **CSF3R** ]  hG CSF R or with a G CSF  [ **CSF3** ]  R  [ **CSF3R** ]  mutant truncated at cytoplasmic amino acid 715.  Ref: 12586631 Blood, 2003
- We also demonstrated that tyrosine phosphorylation of the Src kinases Hck and Lyn occurs following G CSF  [ **CSF3** ]  stimulation of cells expressing WT G CSF  [ **CSF3** ]  R  [ **CSF3R** ] , but that Hck is not phosphorylated in mutant G CSF  [ **CSF3** ]  R  [ **CSF3R** ]  transfectants.  Ref: 12586631 Blood, 2003
- While information exists about the distribution of these isoforms in hematopoietic cells and placenta, G CSF  [ **CSF3** ]  R  [ **CSF3R** ]  isoforms on non hematopoietic fetal tissues have not been described.  Ref: 12660437 Biol Neonate, 2003
- Bone marrow derived primitive CD34 and CD33 CD34 progenitor cells from SCN patients evolving to AML, all with mutations in the granulocyte colony stimulating factor  [ **CSF3** ]  receptor  [ **CSF3R** ]  G CSFR gene, demonstrated normal cell survival.  Ref: 12763135 Exp Hematol, 2003
- Our data provide evidence that the carboxy terminal region of G CSF  [ **CSF3** ]  R  [ **CSF3R** ]  plays a role in the phagocytosis of IgG coated particles.  Ref: 12586631 Blood, 2003
- Thus in the human fetus, G CSF  [ **CSF3** ]  R  [ **CSF3R** ]  isoform I is the predominant form expressed on non hematopoietic and hematopoietic tissues.  Ref: 12660437 Biol Neonate, 2003
- The G CSF  [ **CSF3** ]  R  [ **CSF3R** ]  mutant lacks the immunoreceptor tyrosine based activation motif ITAM, putative binding site for Syk.  Ref: 12586631 Blood, 2003
- In approximately 20% of SCN cases, a truncation mutation is found in the cytoplasmic region of the granulocyte colony stimulating factor  [ **CSF3** ]  receptor  [ **CSF3R** ]  G CSFR.  Ref: 12672695 Blood, 2003
- CD34 cells from 45 patients with myelodysplastic syndrome MDS and MDS acute myeloid leukaemia MDS AML were observed by flow cytometry for the expression of granulocyte colony stimulating factor  [ **CSF3** ]  receptor  [ **CSF3R** ]  G CSFR.  Ref: 12670333 Br J Haematol, 2003
- To this end, we have developed selective amplifier genes SAGs that encode chimeric proteins that are a fusion of granulocyte colony stimulating factor  [ **CSF3** ]  receptor  [ **CSF3R** ]  and the steroid binding domain.  Ref: 12666183 J Gene Med, 2003
- The carboxy terminal region of the granulocyte colony stimulating factor  [ **CSF3** ]  receptor  [ **CSF3R** ]  transduces a phagocytic signal.  Ref: 12586631 Blood, 2003
- IL 10 Milenia Biotec, Bad Nauheim, Germany, gammaINF Diaclone, Besancon, France and G CSF  [ **CSF3** ]  R  [ **CSF3R** ]  &.  Ref: 12788309 Cytokine, 2003
- Different signals are mediated by distinct regions of the cytoplasmic domain of G CSF  [ **CSF3** ]  R  [ **CSF3R** ] , but the precise role of each region has not yet been fully clarified.  Ref: 12586631 Blood, 2003
